# Biomechanical Comparison of Monoplanar and Biplanar Miniplate Fixation for Unfavorable Mandibular Angle Fractures: A Finite Element Analysis

**DOI:** 10.7759/cureus.113865

**Published:** 2026-08-03

**Authors:** Drishti Shah, Shreyas H Gupte, Janhavi Modi, Nitin Jadhav, Shruti Singh, Mayuri Yadav, Richa Agrawal

**Affiliations:** 1 Department of Oral and Maxillofacial Surgery, Dr. G.D. Pol Foundation Y.M.T. Dental College and Hospital, Navi Mumbai, IND; 2 Department of Dentistry, HCG Aastha Cancer Centre, Ahmedabad, IND; 3 Department of Mechanical Engineering, Pillai College of Engineering, Panvel, IND

**Keywords:** biomechanical stability, biplanar plating, finite element analysis, mandibular angle fracture, monoplanar plating

## Abstract

Introduction: Unfavorable mandibular angle fractures are biomechanically challenging because functional masticatory forces may result in displacement of fracture segments and compromise fixation stability. Various plating configurations have been proposed; however, there is limited evidence regarding the biomechanical performance of monoplanar and biplanar plating techniques. This study aimed to compare the biomechanical behaviors of monoplanar and biplanar plating techniques using titanium miniplates with monocortical fixation for the management of unfavorable mandibular angle fractures using finite element analysis.

Materials and methods: A three-dimensional finite element model of an unfavorable mandibular angle fracture was developed using archived computed tomography data. Two fixation configurations were evaluated: monoplanar (Model 1) and biplanar (Model 2) plating. The models were subjected to right unilateral molar clenching (RMOL), left unilateral molar clenching (LMOL), and incisal clenching (INC) tests. von Mises stresses in the plates, screws, and surrounding bone, as well as interfragmentary displacement across the fracture site, were calculated and compared.

Results: The highest plate stress was observed during LMOL in the monoplanar model (546.10 MPa), whereas the biplanar model demonstrated plate stresses of 476.92, 462.90, and 185.76 MPa during RMOL, LMOL, and INC, respectively. Screw stresses were consistently lower in the biplanar model, with values of 51.00, 43.34, and 24.47 MPa compared with 149.28, 235.89, and 53.32 MPa in the monoplanar model during RMOL, LMOL, and INC, respectively. Bone stress values were also reduced in the biplanar model (59.37, 110.32, and 38.32 MPa) compared with those in the monoplanar model (227.20, 137.03, and 63.72 MPa). Interfragmentary displacement was lower in the biplanar model under all loading conditions, ranging from 18.86 to 30.41 μm compared with 22.98 to 38.20 μm in the monoplanar model.

Conclusion: Biplanar plating demonstrated lower screw stress, reduced bone stress, and decreased interfragmentary displacement compared with monoplanar plating, indicating superior biomechanical stability for the fixation of unfavorable mandibular angle fractures.

## Introduction

Mandibular fractures are among the most common maxillofacial injuries encountered in clinical practice, with the angle of the mandible being one of the most frequently affected anatomical sites [1]. The mandibular angle is particularly susceptible to fracture owing to the abrupt transition between the mandibular body and ramus, reduced cross-sectional bone thickness, and frequent presence of impacted third molars [2]. Unfavorable mandibular angle fractures present an additional challenge because the direction of the fracture line and the action of masticatory muscles, including the masseter, temporalis, and medial pterygoid, tend to displace the fracture segments, compromising stability and increasing the risk of postoperative complications [3].

Open reduction and internal fixation (ORIF) using titanium miniplates is the standard treatment for mandibular angle fractures [4]. Since the introduction of Champy’s principles of osteosynthesis, various plating configurations have been proposed to achieve optimal biomechanical stability while minimizing surgical complications. Although single superior-border miniplate fixation remains widely accepted, concerns regarding inferior border gapping, screw loosening, plate deformation, and fracture instability have prompted the development of alternative fixation strategies, including the use of two miniplates in different orientations. The biomechanical behavior of these plating systems remains a subject of ongoing debate, particularly in the management of unfavorable fracture patterns [5,6].

Finite element analysis (FEA) has emerged as a reliable computational tool for evaluating stress distribution, strain patterns, and displacement within complex biological structures under simulated physiological loading conditions. By providing detailed insights into the interaction between bone and fixation hardware, FEA enables an objective assessment of fixation stability without the limitations associated with clinical or cadaveric studies [7,8]. Therefore, the present in vitro FEA study was undertaken to comparatively evaluate monoplanar and biplanar plating techniques using titanium miniplates with monocortical fixation for the treatment of unfavorable mandibular angle fractures. The objectives were to assess the stress distribution within the plates and screws, evaluate the stress transfer to the surrounding bone, determine the potential risk of plate failure and screw loosening, and compare the interfragmentary displacement between the two fixation techniques to identify the biomechanically more stable plating configuration.

This article is based on the dissertation submitted by DS to the Maharashtra University of Health Sciences, Nashik, in partial fulfillment of the requirements for the MDS degree in Oral and Maxillofacial Surgery (2020-2023), under the guidance of SHG.

## Materials and methods

This in vitro FEA study was conducted at the Department of Oral and Maxillofacial Surgery, Dr. G.D. Pol Foundation Y.M.T. Dental College and Hospital, Navi Mumbai, India, as part of the first author's postgraduate (MDS) dissertation registered with the Maharashtra University of Health Sciences, Nashik (2020-2023). Finite element modeling and analysis were completed in February 2023 using ANSYS 2020 R1; the figures present the outputs of that analysis. The manuscript was prepared subsequently. This computational study used a single anonymized archived CT scan and involved no patient participation. The Institutional Ethics Committee of Dr. G.D. Pol Foundation YMT Dental College and Hospital determined (Ref. YMTDC/IEC/OUT/2026/1-EW, 27 August 2026) that the use of the archived scan was covered by a waiver under institutional policy for secondary use of archived imaging data.

A computed tomography (CT) scan of a dentate adult patient with an unfavorable mandibular angle fracture was retrieved from the departmental archival records and used for the model generation. The scans were selected based on predefined criteria, which included the presence of a unilateral unfavorable mandibular angle fracture, complete permanent dentition, adequate image quality, and absence of significant artifacts. Scans demonstrating partial or complete edentulism, gross mandibular asymmetry, pathological lesions, congenital craniofacial deformities, previous mandibular surgery, healed or malunited fractures, or inadequate image quality were excluded.

Digital Imaging and Communications in Medicine (DICOM) data were imported into MIMICS software (version 21.0, Materialise NV, Leuven, Belgium) for segmentation and three-dimensional reconstruction of the fractured mandible. The reconstructed model was exported in stereolithography (STL) format and further processed in Rhinoceros 3D software (Robert McNeel & Associates, Seattle, WA, USA) for the virtual reduction of the fracture and refinement of anatomical details. Titanium miniplates and monocortical screws were digitally designed and assembled using SOLIDWORKS 2020 software (Dassault Systèmes, Vélizy-Villacoublay, France).

**Figure 1:**
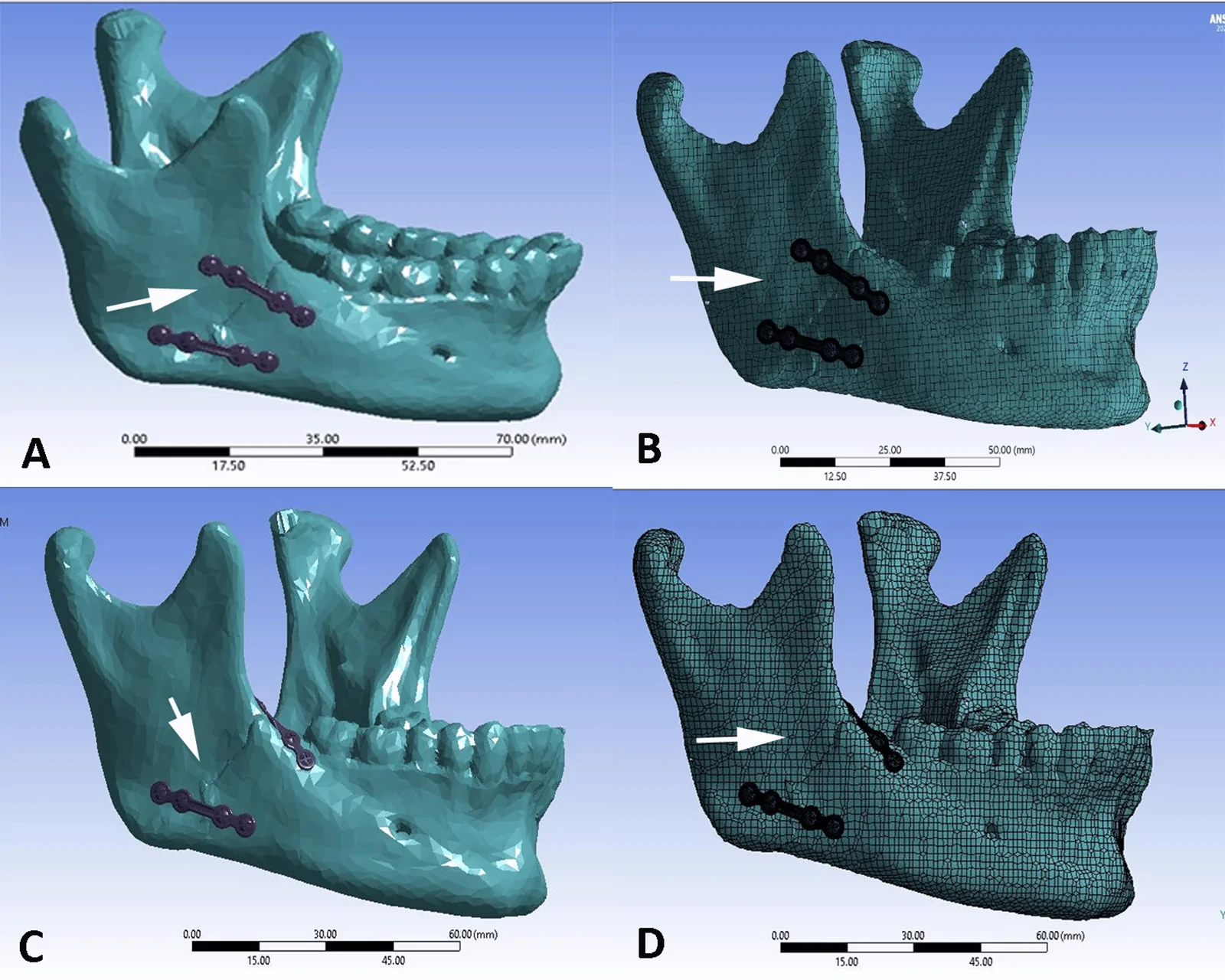
Finite element models used in the study: (A) monoplanar plating model (Model 1) showing two parallel miniplates positioned along the lateral surface of the mandibular angle, (B) meshed finite element representation of the monoplanar model, (C) biplanar plating model (Model 2) showing one superior border miniplate and one inferior border miniplate, and (D) meshed finite element representation of the biplanar model. Arrows indicate the fixation systems evaluated. All images were generated directly using the ANSYS software (ANSYS Inc., Canonsburg, PA, USA) in high-definition resolution.

Two fixation models were created for this study. Model 1 represented a monoplanar plating configuration (Figures 1A, 1B) consisting of two parallel four-hole titanium miniplates positioned along the lateral surface of the mandibular angle. Model 2 represented a biplanar plating configuration (Figures 1C, 1D) in which one four-hole titanium miniplate was positioned along the superior border, following Champy’s ideal line of osteosynthesis, and a second miniplate was placed at the inferior border of the mandible. Both fixation systems used Grade V titanium alloy miniplates and monocortical screws.

The geometric models were imported into ANSYS 2020 R1 (ANSYS Inc., Canonsburg, PA, USA) for the FEA. Meshing was performed using a combination of tetrahedral and hexahedral elements with an average element size of 1.5 mm. Mesh convergence testing was performed to ensure the numerical stability and accuracy of the computational model. The mandibular cortical bone, titanium plates, and screws were assumed to be homogeneous, isotropic, and linearly elastic. The material properties were assigned to each structure. Young’s modulus and Poisson’s ratio values used for the cortical bone and titanium alloy are presented in Table 1.

**Table 1:** Material properties used in the finite element model. MPa: megapascals; Ti-6Al-4V: titanium-6 aluminum-4 vanadium

Material	Young’s modulus (MPa)	Poisson’s ratio
Cortical bone	14,800	0.30
Titanium alloy (Ti-6Al-4V)	105,000	0.33

**Figure 2:**
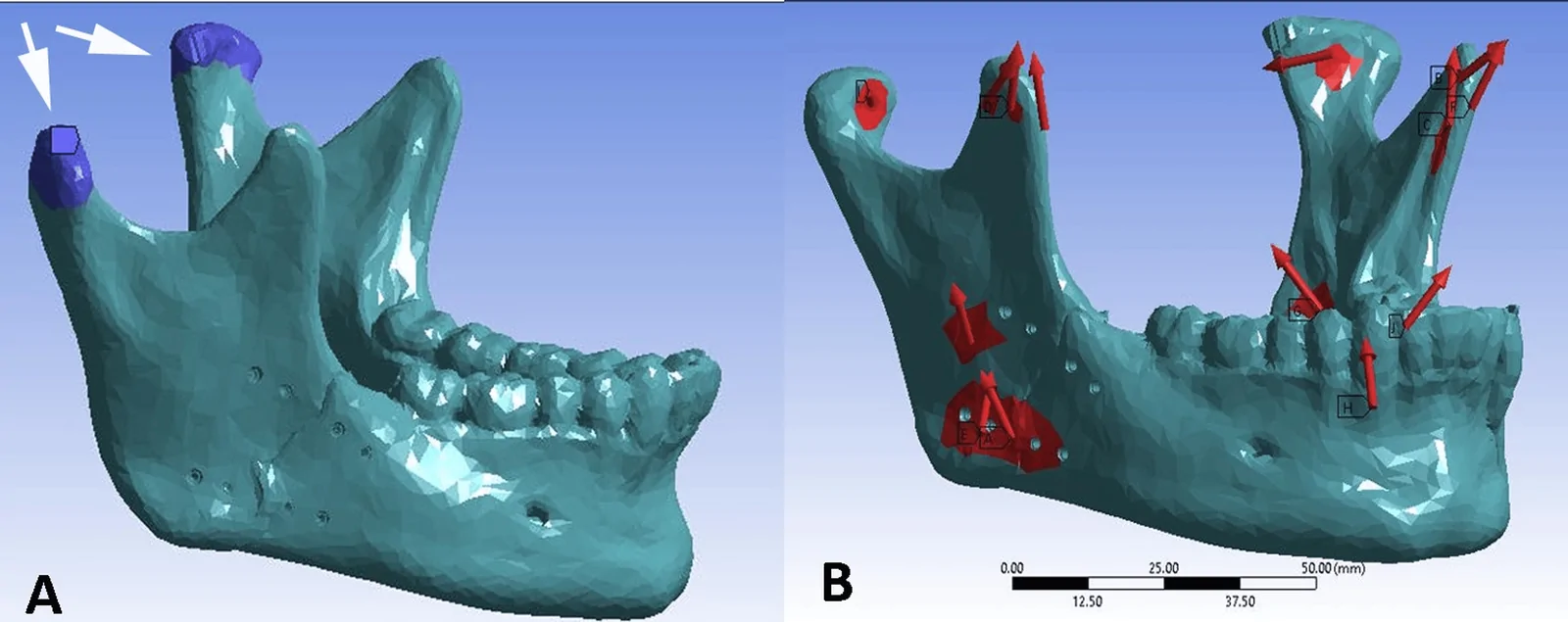
Boundary conditions and muscle force application: (A) fixed constraints applied at the superior surfaces of both mandibular condyles; (B) anatomical sites and directions of masticatory muscle forces used in the finite element model. The vectors represent the superficial masseter, deep masseter, medial pterygoid, anterior temporalis, middle temporalis, posterior temporalis, and inferior lateral pterygoid muscles. All images were generated directly using the ANSYS software (ANSYS Inc., Canonsburg, PA, USA) in high-definition resolution.

Boundary conditions were established to simulate the physiological function of the mandible. The superior surfaces of both mandibular condyles were constrained in all directions to reproduce the articulation within the temporomandibular joints and prevent rigid body motion (Figure 2A). A bonded contact condition was assumed between the plates, screws, and surrounding bone to simulate the rigid fixation. Muscle forces corresponding to the superficial masseter, deep masseter, medial pterygoid, anterior temporalis, middle temporalis, posterior temporalis, and inferior lateral pterygoid muscles were applied to the model. The origin, magnitude, and direction of these muscle forces were incorporated into the finite element model to replicate the physiological loading conditions (Figure 2B).

**Figure 3:**
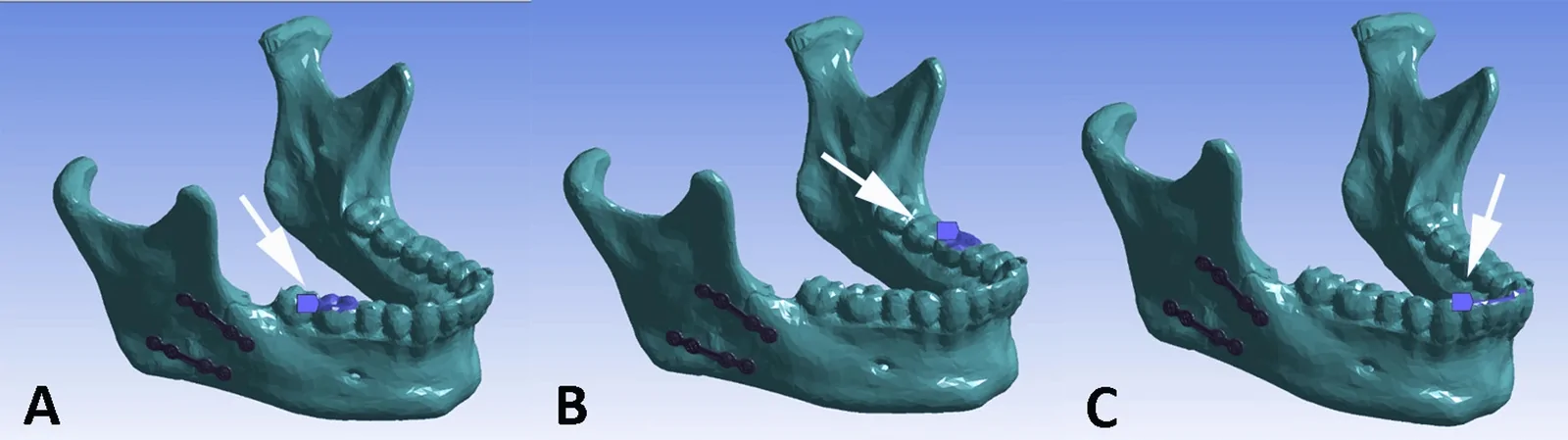
Functional loading conditions applied to the finite element models: (A) right unilateral molar clenching (RMOL), (B) left unilateral molar clenching (LMOL), and (C) incisal clenching (INC). Arrows indicate the constrained occlusal contact regions used to simulate masticatory loading. All images were generated directly using the ANSYS software (ANSYS Inc., Canonsburg, PA, USA) in high-definition resolution.

Three functional loading conditions were simulated for each fixation model: right unilateral molar clenching (RMOL) (Figure 3A), left unilateral molar clenching (LMOL) (Figure 3B), and incisal clenching (INC) (Figure 3C). For RMOL, the right first molar region was constrained in the vertical direction; for LMOL, the left first molar region was constrained; and for INC, the incisor region was constrained in the vertical direction.

These loading conditions were selected to reproduce the common functional occlusal scenarios encountered during mastication (Tables 2-4).

**Table 2:** Magnitude and vector components of muscle forces (N) applied during right unilateral molar clenching (RMOL). Mag R: force magnitude on the right side (N); Mag L: force magnitude on the left side (N); Fx: force component along the x-axis (N); Fy: force component along the y-axis (N); Fz: force component along the z-axis (N); R: right; L: left Data presented in Newton (N).

Muscle	Mag R	Mag L	Fx R	Fy R	Fz R	Fx L	Fy L	Fz L
Superficial masseter	137.1	114.2	–28.4	57.4	121.2	23.6	47.9	101.0
Deep masseter	58.8	49.0	–32.1	–21.0	44.5	26.7	–17.5	37.1
Medial pterygoid	146.8	104.9	71.4	54.8	116.1	–51.0	39.1	83.0
Anterior temporalis	115.3	91.6	–17.2	5.1	114.0	13.7	4.0	90.5
Middle temporalis	63.1	64.1	–14.0	–31.5	52.8	14.2	–32.0	53.6
Posterior temporalis	44.6	29.5	–9.3	–38.1	21.1	6.1	–25.2	14.0
Inferior lateral pterygoid	20.1	43.5	12.6	15.2	–3.5	–27.4	32.9	–7.6

**Table 3:** Magnitude and vector components of muscle forces (N) applied during left unilateral molar clenching (LMOL). Mag R: force magnitude on the right side (N); Mag L: force magnitude on the left side (N); Fx: force component along the x-axis (N); Fy: force component along the y-axis (N); Fz: force component along the z-axis (N); R: right; L: left Data presented in Newton (N).

Muscle	Mag R	Mag L	Fx R	Fy R	Fz R	Fx L	Fy L	Fz L
Superficial masseter	114.2	137.1	–23.6	47.8	101.0	28.4	57.4	121.2
Deep masseter	49.0	58.8	–26.8	–17.5	37.1	32.1	–21.1	44.6
Medial pterygoid	104.9	146.9	51.0	39.1	83.0	–71.4	54.8	116.2
Anterior temporalis	91.7	115.4	–13.7	4.0	90.6	17.2	5.1	114.0
Middle temporalis	64.0	63.1	–14.2	–32.0	53.6	14.0	–31.6	52.8
Posterior temporalis	29.5	44.6	–6.1	–25.2	14.0	9.3	–38.1	21.1
Inferior lateral pterygoid	43.5	20.1	27.4	32.9	–7.6	–12.7	15.2	–3.5

**Table 4:** Magnitude and vector components of muscle forces (N) applied during incisal clenching (INC). Mag R: force magnitude on the right side (N); Mag L: force magnitude on the left side (N); Fx: force component along the x-axis (N); Fy: force component along the y-axis (N); Fz: force component along the z-axis (N); R: right; L: left Data presented in Newton (N).

Muscle	Mag R	Mag L	Fx R	Fy R	Fz R	Fx L	Fy L	Fz L
Superficial masseter	76.2	76.2	–15.8	31.9	67.3	15.8	31.9	67.3
Deep masseter	21.2	21.2	–11.6	–7.6	16.1	11.6	–7.6	16.1
Medial pterygoid	136.3	136.3	66.3	50.9	107.8	–66.3	50.9	107.8
Anterior temporalis	12.6	12.6	–1.9	0.6	12.5	1.9	0.6	12.5
Middle temporalis	5.7	5.7	–1.3	–2.9	4.8	1.3	–2.9	4.8
Posterior temporalis	3.0	3.0	–0.6	–2.6	1.4	0.6	–2.6	1.4
Inferior lateral pterygoid	47.5	47.5	29.9	36.0	–8.3	–29.9	36.0	–8.3

The biomechanical performance of the two plating techniques was evaluated by determining the von Mises stress distribution within the plates, screws, and surrounding mandibular bone. The maximum von Mises stress values within the fixation hardware were used to assess the risk of plate deformation and screw loosening. The stress distribution within the cortical bone was analyzed to evaluate the load transfer characteristics and biomechanical behavior of each fixation system. In addition, the displacement across the fracture line at the inferior border of the mandible was measured to determine the interfragmentary movement and fixation stability. The stress and displacement patterns generated under each loading condition were compared between the monoplanar and biplanar fixation models to identify the biomechanically superior plating configuration for managing unfavorable mandibular angle fractures.

## Results

The finite element models were successfully analyzed under three functional loading conditions: RMOL, LMOL, and INC. The maximum von Mises stresses generated within the plates, screws, and surrounding bone, together with the interfragmentary displacement values, are listed in Table 5.

**Table 5:** Comparison of maximum von Mises stresses and interfragmentary displacement between monoplanar and biplanar plating models under different loading conditions. RMOL: right unilateral molar clenching; LMOL: left unilateral molar clenching; INC: incisal clenching; MPa: megapascal; μm: micrometer

Parameter	Model	RMOL	LMOL	INC
Plate stress (MPa)	Monoplanar plating	413.22	546.10	158.83
Plate stress (MPa)	Biplanar plating	476.92	462.90	185.76
Screw stress (MPa)	Monoplanar plating	149.28	235.89	53.32
Screw stress (MPa)	Biplanar plating	51.00	43.34	24.47
Bone stress (MPa)	Monoplanar plating	227.20	137.03	63.72
Bone stress (MPa)	Biplanar plating	59.37	110.32	38.32
Displacement (μm)	Monoplanar plating	38.20	28.91	22.98
Displacement (μm)	Biplanar plating	30.41	20.10	18.86

**Figure 4:**
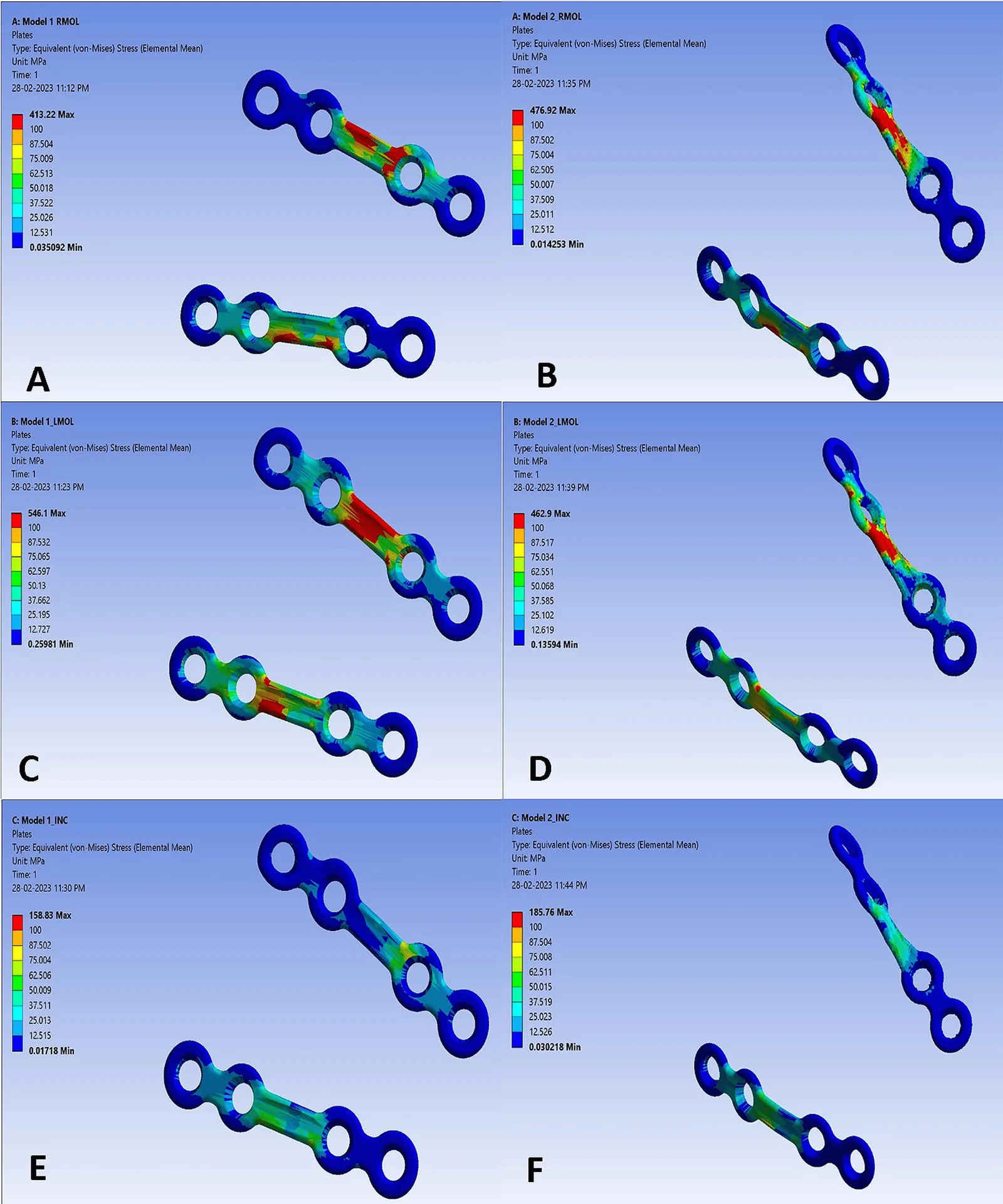
von Mises stress distribution within fixation plates under different loading conditions: (A) monoplanar model during RMOL, (B) biplanar model during RMOL, (C) monoplanar model during LMOL, (D) biplanar model during LMOL, (E) monoplanar model during INC, and (F) biplanar model during INC. Stress values are expressed in MPa. All images were generated directly using the ANSYS software (ANSYS Inc., Canonsburg, PA, USA) in high-definition resolution. RMOL: right unilateral molar clenching; LMOL: left unilateral molar clenching; INC: incisal clenching

For the fixation plates, the highest von Mises stress was observed during LMOL in the monoplanar plating model (546.10 MPa). During RMOL and INC, the biplanar plating model demonstrated higher plate stress values of 476.92 and 185.76 MPa, respectively, compared with 413.22 and 158.83 MPa in the monoplanar model, respectively. However, under all loading conditions, the recorded stress values remained below the yield strength of the Grade V titanium alloy. Stress concentrations were predominantly located in the central region of the plates and were more pronounced in the superior plate positioned along the external oblique ridge in the biplanar configuration (Figure 4).

**Figure 5:**
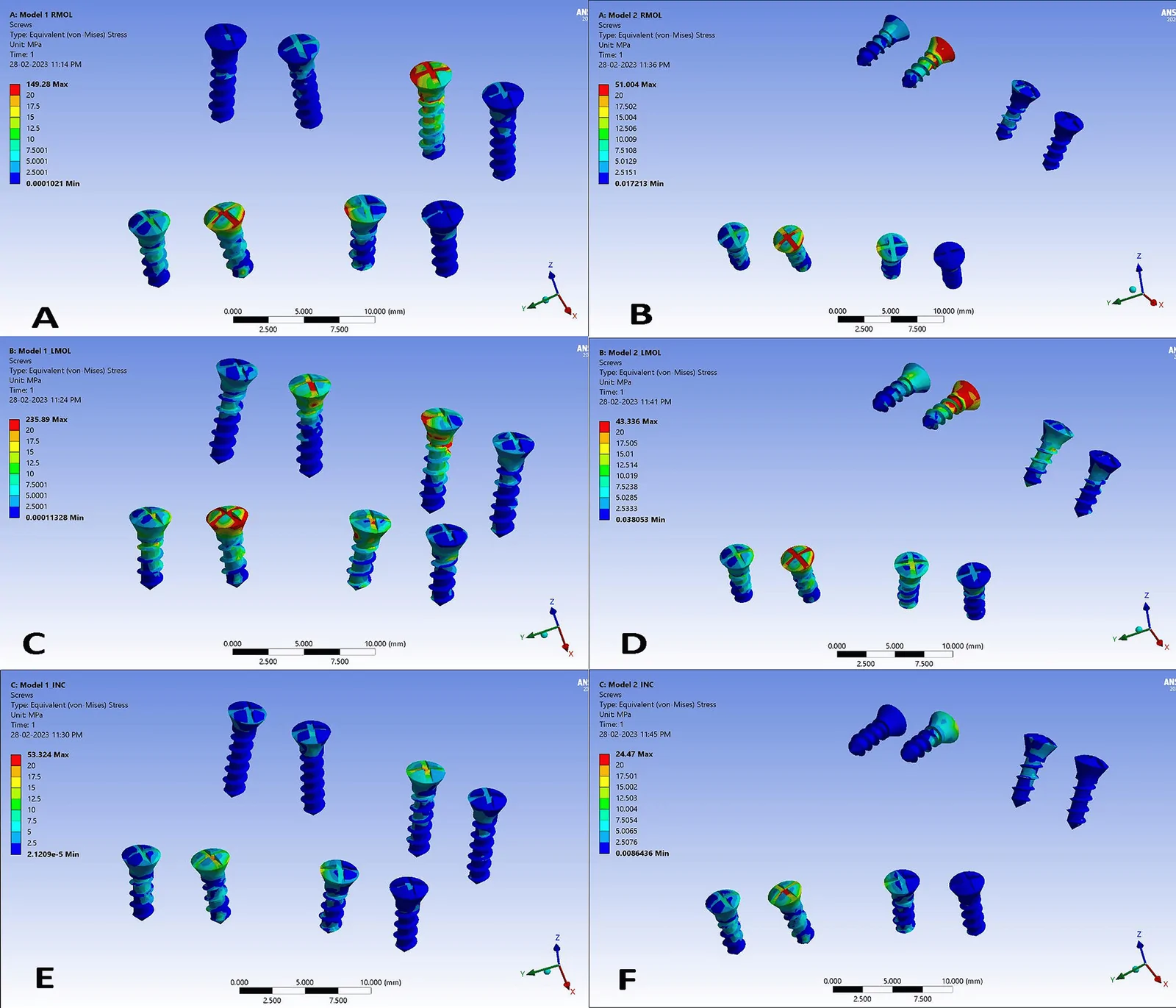
von Mises stress distribution within fixation screws under different loading conditions: (A) monoplanar model during RMOL, (B) biplanar model during RMOL, (C) monoplanar model during LMOL, (D) biplanar model during LMOL, (E) monoplanar model during INC, and (F) biplanar model during INC. Stress values are expressed in MPa. All images were generated directly using the ANSYS software (ANSYS Inc., Canonsburg, PA, USA) in high-definition resolution. RMOL: right unilateral molar clenching; LMOL: left unilateral molar clenching; INC: incisal clenching

The screws in the monoplanar plating model consistently exhibited higher von Mises stress values than those in the biplanar model for all loading conditions. During RMOL, LMOL, and INC, the screw stresses in the monoplanar model were 149.28, 235.89, and 53.32 MPa, respectively, compared with 51.00, 43.34, and 24.47 MPa in the biplanar model. The highest screw stress was recorded during LMOL in the monoplanar model group. Stress concentrations were mainly localized in the screws closest to the fracture line (Figure 5).

**Figure 6:**
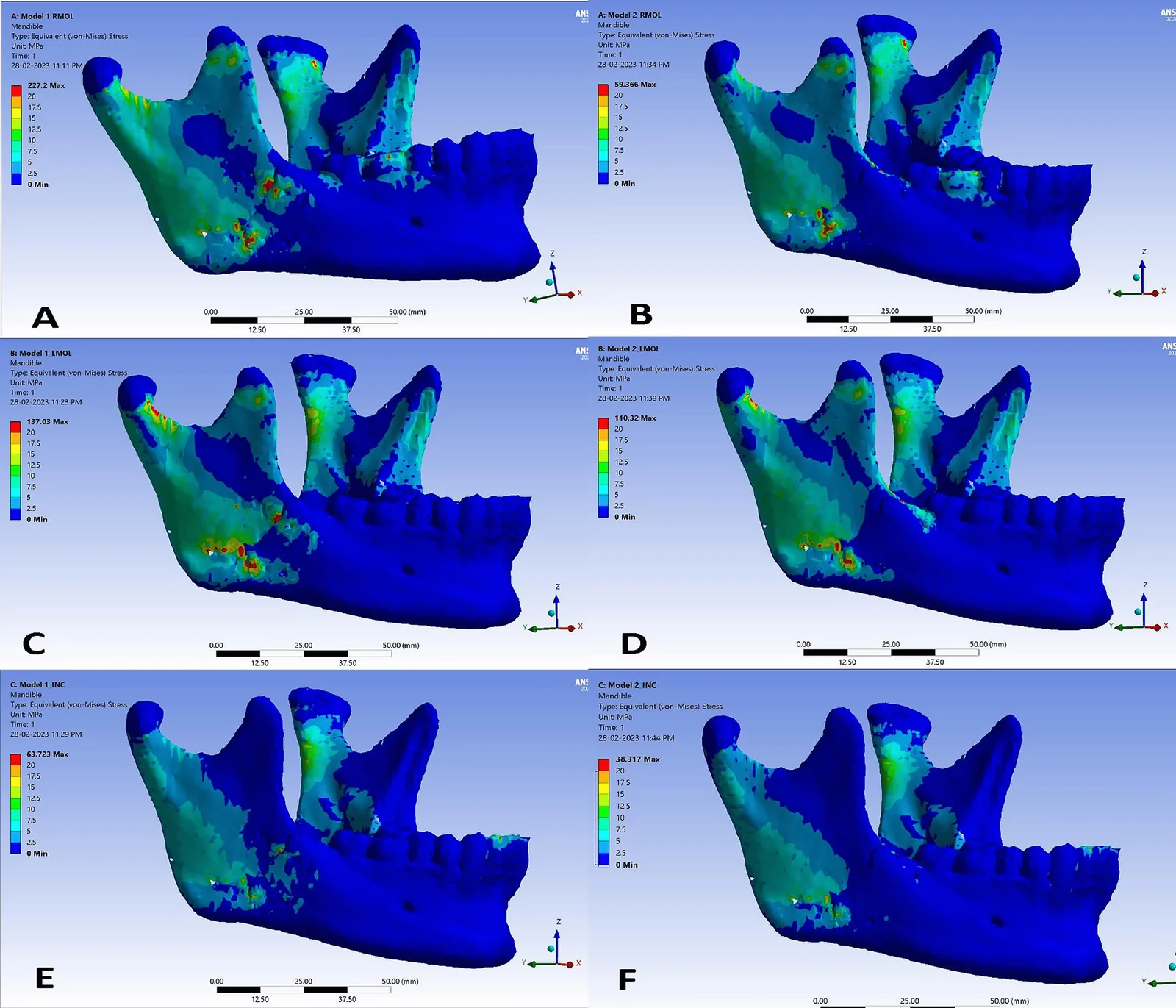
von Mises stress distribution within mandibular bone under different loading conditions: (A) monoplanar model during RMOL, (B) biplanar model during RMOL, (C) monoplanar model during LMOL, (D) biplanar model during LMOL, (E) monoplanar model during INC, and (F) biplanar model during INC. Stress values are expressed in MPa. All images were generated directly using the ANSYS software (ANSYS Inc., Canonsburg, PA, USA) in high-definition resolution. RMOL: right unilateral molar clenching; LMOL: left unilateral molar clenching; INC: incisal clenching

The stress distribution within the mandibular bone differed between the two fixation techniques. The monoplanar model demonstrated higher bone stress under all loading conditions, with values of 227.20 MPa during RMOL, 137.03 MPa during LMOL, and 63.72 MPa during INC. The corresponding values in the biplanar model were substantially lower at 59.37, 110.32, and 38.32 MPa, respectively. Figure 6 shows the stress distribution patterns within the mandibular bone. In both fixation models, stress concentrations were primarily observed around the fracture line and in the adjacent cortical bone. However, the biplanar configuration exhibited a more uniform stress distribution with reduced peak stress concentrations than the monoplanar configuration.

**Figure 7:**
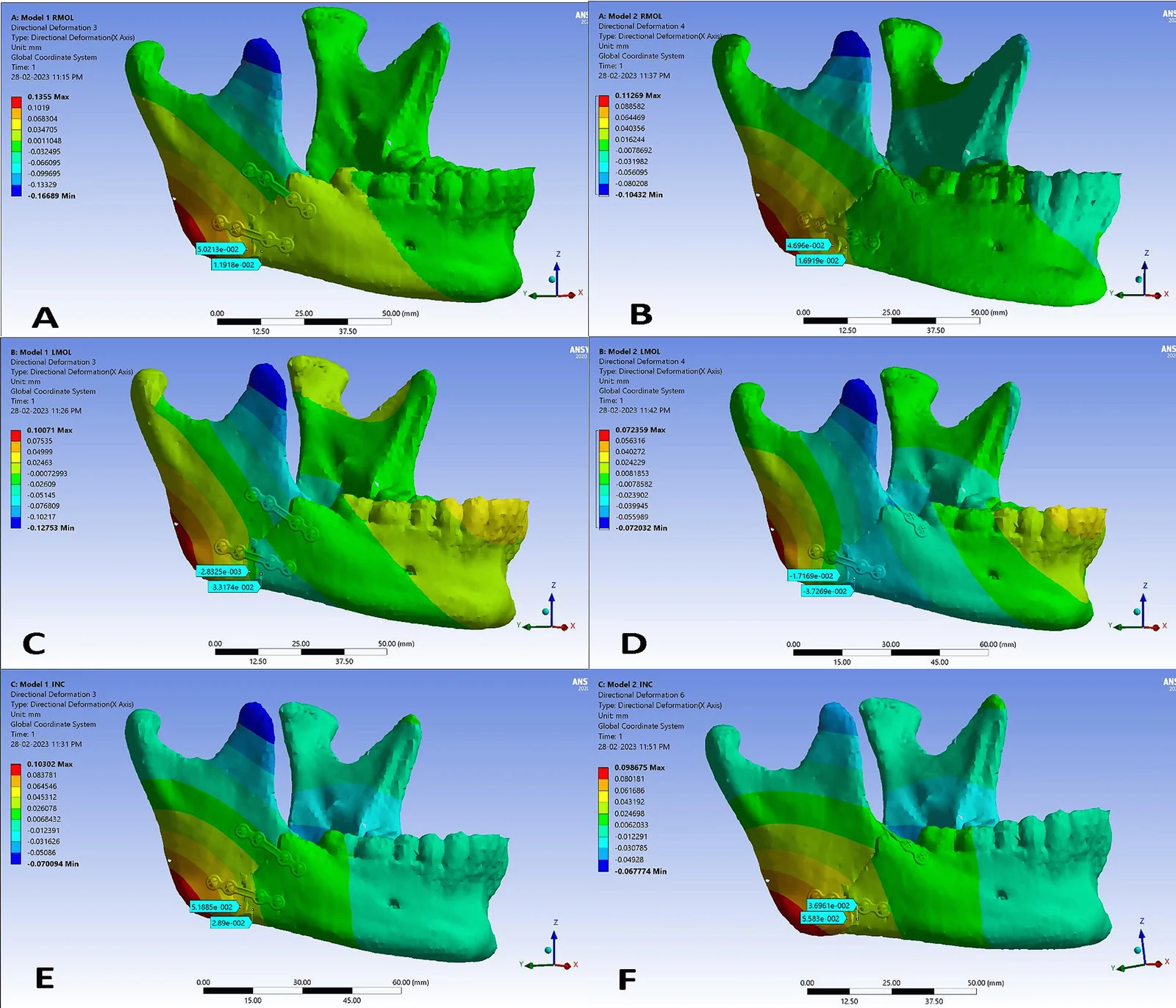
Directional displacement at the inferior border of the mandible under different loading conditions: (A) monoplanar model during RMOL, (B) biplanar model during RMOL, (C) monoplanar model during LMOL, (D) biplanar model during LMOL, (E) monoplanar model during INC, and (F) biplanar model during INC. Displacement values are expressed in μm. All images were generated directly using the ANSYS software (ANSYS Inc., Canonsburg, PA, USA) in high-definition resolution. RMOL: right unilateral molar clenching; LMOL: left unilateral molar clenching; INC: incisal clenching

Interfragmentary displacement at the inferior border of the mandible was lower in the biplanar plating model under all loading conditions. During RMOL, LMOL, and INC, the displacement values in the monoplanar model were 38.20, 28.91, and 22.98 μm, respectively, whereas the corresponding values in the biplanar model were 30.41, 20.10, and 18.86 μm. The greatest displacement was observed during the RMOL in both models. The reduced displacement values recorded in the biplanar model indicated improved biomechanical stability across the fracture site under functional loading (Figure 7).

Overall, the biplanar plating technique demonstrated lower screw stress, lower bone stress, and reduced interfragmentary displacement than the monoplanar plating technique. Although higher plate stresses were observed in the biplanar model during the RMOL and INC, all stress values remained within the acceptable mechanical limits of the titanium fixation system.

## Discussion

The management of unfavorable mandibular angle fractures remains a subject of considerable debate because of the complex biomechanical environment generated by masticatory forces and the tendency of the proximal and distal segments to undergo displacement during function [9]. The present FEA compared the biomechanical performance of monoplanar and biplanar plating techniques using titanium miniplates with monocortical fixation under three clinically relevant loading conditions: RMOL, LMOL, and INC. The findings demonstrated that the biplanar plating configuration resulted in lower screw and bone stresses and reduced interfragmentary displacement compared with the monoplanar plating technique, indicating superior biomechanical stability.

One of the primary objectives of this study was to evaluate the stress distribution within the fixation plates. The highest plate stress was observed in the monoplanar model during LMOL, whereas under the RMOL and INC conditions, the biplanar model demonstrated higher plate stresses. Importantly, none of the recorded values exceeded the yield strength of Grade V titanium alloy, suggesting that both fixation systems can withstand physiological loading without mechanical failure [10]. The increased stress concentration observed within the superior plate of the biplanar configuration may be attributed to the redistribution of forces through a more rigid three-dimensional framework, whereby the fixation hardware absorbs a greater proportion of the applied load, thereby protecting the surrounding bone and screw. Similar findings were reported by Alkan et al. [11], who demonstrated that dual-plate fixation systems exhibit more favorable biomechanical behavior than conventional single-plane fixation because of improved resistance to bending and torsional forces.

Chrcanovic [12] reviewed the available biomechanical and computer-based studies on mandibular angle fracture fixation and reported that biplanar miniplate orientation provides greater biomechanical stability than monoplanar fixation. The author further noted that the use of two miniplates reduced inferior border gapping and fracture displacement, findings that are consistent with the reduced bone stress and interfragmentary displacement observed in the present FEA. Ponvel et al. [13] reported that strategic modification of plate orientation improved the stress distribution and reduced fracture fragment displacement. Similarly, the biplanar plating configuration in the present study exhibited lower bone stress and reduced interfragmentary displacement than the monoplanar model, indicating enhanced biomechanical stability.

Assessment of screw stresses revealed substantially lower von Mises stress values in the biplanar model under all the loading conditions. The concentration of stress around screws adjacent to the fracture line observed in the present study is consistent with the findings of Lovald et al. [14], who reported that screws located nearest to the fracture site are subjected to the highest functional loads. Lower screw stress is clinically advantageous because it reduces the likelihood of screw loosening, micromotion, and eventual fixation failure. The marked reduction in screw stress observed with the biplanar technique suggests that load sharing occurred more effectively between the two plates, thereby decreasing the stress concentration at individual fixation points.

Another important finding was the difference in stress transfer to the mandibular bone. The monoplanar model consistently exhibited greater bone stress than the biplanar model under all the loading conditions. Bone stress during the RMOL was particularly high in the monoplanar configuration compared to that in the biplanar configuration. Similar reductions were observed during the LMOL and INC. These findings indicate that the biplanar arrangement provides more effective stress dissipation across the mandibular angle region. From a biomechanical perspective, excessive stress concentration within the bone may contribute to localized bone resorption, delayed healing, or instability at the fracture site. Schierle et al. [15] demonstrated that the addition of a second miniplate at the inferior border improves force distribution and reduces unfavorable stress concentration around the fracture. Likewise, Khiabani et al. [16] reported that double miniplate fixation in different planes provided better biomechanical performance and higher functional bite force than single plate fixation in mandibular angle fractures. These findings corroborate the present FEA, in which the biplanar plating model demonstrated reduced bone stress, lower screw stress, and decreased interfragmentary displacement.

Interfragmentary displacement is one of the most critical indicators of fixation stability because excessive movement at the fracture interface can compromise bone healing. In the present study, the displacement values were consistently lower in the biplanar model under all loading conditions. These findings suggest that biplanar plating offers superior resistance to functional loading and better maintenance of fracture segment alignment. Kimsal et al. [17] reported that the combined use of a superior tension band and inferior compression plate resulted in the lowest plate stress and fracture site strain among the evaluated fixation methods. Similarly, the biplanar model in the present study exhibited a more favorable stress distribution and reduced interfragmentary displacement than the monoplanar configuration. The three-dimensional stability achieved by fixation in two planes appears to be responsible for the reduced displacement observed in this study.

The observed stress patterns also provide insights into the biomechanical behavior of the two fixation techniques. In both models, stress concentrations were primarily localized around the fracture line and adjacent to the cortical bone. However, the biplanar model demonstrated a more uniform distribution of stress throughout the mandibular angle region than the monoplanar model. This finding supports the concept that fixation in two planes creates a stable framework capable of neutralizing tensile, compressive, and torsional forces more effectively than fixation in a single plane. Choi et al. [18] reported that double miniplate fixation produced significantly greater biomechanical stability than single miniplate fixation in atrophic mandibular fracture.

Clinical implications

The findings of this study suggest that biplanar plating may provide superior biomechanical stability for the management of unfavorable mandibular angle fractures. Lower screw stress, reduced bone stress, and decreased interfragmentary displacement may contribute to improved fracture stabilization and potentially reduce the risk of fixation failure, screw loosening, and postoperative segment mobility. Although both fixation techniques remained within the mechanical limits of titanium alloy hardware, the biplanar configuration demonstrated a more favorable biomechanical profile and may therefore be considered when enhanced rigidity is required.

Limitations

This study had several limitations. FEA is a computational simulation that cannot completely replicate the biological complexity of in vivo conditions. The bone was assumed to be homogeneous, isotropic, and linearly elastic, whereas the actual mandibular bone exhibits anisotropic and heterogeneous characteristics. Soft tissues, periodontal ligament properties, muscular adaptation, and bone remodeling processes were not incorporated into the model. Furthermore, only one fracture configuration and three clenching conditions were evaluated in this study. Future studies should investigate different fracture patterns, alternative fixation designs, cyclic loading conditions, and patient-specific models to further validate the biomechanical performance of these fixation techniques.

## Conclusions

Within the limitations of this FEA, the biplanar plating technique demonstrated superior biomechanical performance compared with the monoplanar plating technique for the fixation of unfavorable mandibular angle fractures. Biplanar fixation resulted in lower screw stresses, reduced bone stresses, and decreased interfragmentary displacement under all clenching conditions, indicating enhanced fracture stability and a more favorable load distribution. Although both fixation methods remained within the mechanical limits of titanium hardware, biplanar plating provided a more stable fixation framework and may be considered a preferable option when increased rigidity and resistance to functional loading are required.
