# Correction: Biomechanical Comparison of Monoplanar and Biplanar Miniplate Fixation for Unfavorable Mandibular Angle Fractures: A Finite Element Analysis

**DOI:** 10.7759/cureus.c493

**Published:** 2026-10-16

**Authors:** Drishti Shah, Shreyas H Gupte, Janhavi Modi, Nitin Jadhav, Shruti Singh, Mayuri Yadav, Richa Agrawal

**Affiliations:** 1 Department of Oral and Maxillofacial Surgery, Dr. G.D. Pol Foundation Y.M.T. Dental College and Hospital, Navi Mumbai, IND; 2 Department of Dentistry, HCG Aastha Cancer Centre, Ahmedabad, IND; 3 Department of Mechanical Engineering, Pillai College of Engineering, Panvel, IND

Following a reader inquiry and editorial review, this article has been corrected. The analysis and figures are unchanged and the conclusions are unaffected.

The study was performed in February 2023 as part of the first author's MDS dissertation at the Maharashtra University of Health Sciences, Nashik, not between June 2024 and December 2025 as originally stated; the Methods have been corrected and a dissertation statement added to the Introduction.

Table 1 now gives the material values used in the analysis (cortical bone 14,800 MPa and 0.30; titanium alloy 105,000 MPa and 0.33). Five stress values in Table 5 have been corrected to match the figure legends, with the Abstract and Results revised accordingly.

The ethics statement now cites the Institutional Ethics Committee's waiver determination (Ref. YMTDC/IEC/OUT/2026/1-EW, 27 August 2026).

Dr. Aniket A. Bonde has been moved from the author list to the Acknowledgements with his agreement, as his contributions did not meet the ICMJE authorship criteria; the Author Contributions and Acknowledgements have been revised with the consent of those named.

In the Discussion, "panoramic model" has been corrected to "monoplanar model".

